# Knowledge, attitudes, information sources, and use of weight-loss injections among adults in Al-Ahsa, Saudi Arabia: a cross-sectional study

**DOI:** 10.3389/fpubh.2026.1918350

**Published:** 2026-09-23

**Authors:** Shahad Alshamrani, Fadwa Alqasem, Danah Alkhamis, Munerah Almulhem

**Affiliations:** Department of Public Health, King Faisal University, Al Ahsa, Saudi Arabia

**Keywords:** GLP-1 receptor agonists, medication literacy, obesity, Saudi Arabia, semaglutide, social media, tirzepatide, weight-loss injections

## Abstract

**Background:**

The growing use and public visibility of injectable weight-loss medications, including GLP-1 receptor agonists and related therapies, have raised important public health concerns regarding knowledge, attitudes, information sources, and safe use. This study assessed knowledge, attitudes, information sources, perceived social media influence, misconceptions, and self-reported use or consideration of weight-loss injections among adults in Al-Ahsa, Saudi Arabia, and examined associations with selected participant characteristics and BMI.

**Methods:**

This descriptive, cross-sectional study included 718 adults in Al-Ahsa, Saudi Arabia, and was conducted using an online structured questionnaire that was reviewed by experts and pilot-tested before the main data collection. Data were analyzed using the Statistical Package for the Social Sciences version 29. Descriptive statistics were used to summarize participant characteristics, knowledge, attitudes, information sources, perceived social media influence, and use patterns. Chi-square tests were used to examine associations between weight-loss injection use or consideration and selected participant characteristics.

**Results:**

Knowledge responses showed uncertainty regarding several aspects of weight-loss injections, particularly their mechanisms and potential adverse effects. Participants generally recognized the risks associated with unsupervised use, while perceptions regarding effectiveness and long-term safety were more varied. Overall, 8.5% of participants reported current or previous use of weight-loss injections, and 8.5% reported considering future use. Among users, tirzepatide was the most commonly reported injection (42.6%), followed by semaglutide (39.3%) and liraglutide (14.8%). Social media was the most frequently reported source of information (62.1%), followed by friends or family (41.5%) and healthcare professionals (39.1%). Use or consideration of weight-loss injections was significantly associated with age group, education level, and BMI category, but not sex.

**Conclusion:**

Adults in Al-Ahsa showed considerable uncertainty across several knowledge items and generally mixed perceptions regarding the effectiveness and safety of weight-loss injections, with relatively low current or previous use but emerging interest in future use.

## Background

Obesity is a major public health concern worldwide and is associated with increased risk of non-communicable diseases, premature mortality, and healthcare burden (1, 2). According to the World Health Organization, 2.5 billion adults were overweight in 2022, including 890 million adults living with obesity. Obesity also represents an important public health challenge in Saudi Arabia; recent national data showed that 23.1% of individuals aged 15 years and above were classified as obese, and 45.1% as overweight (3, 4).

In response to the high burden of obesity, pharmacological treatments have become increasingly important as part of comprehensive weight-management care. Injectable medications, including glucagon-like peptide-1 (GLP-1) receptor agonists such as semaglutide and liraglutide, as well as the dual glucose-dependent insulinotropic polypeptide and GLP-1 receptor agonist tirzepatide, have demonstrated clinically meaningful weight reduction in adults with overweight or obesity when used alongside lifestyle modification (5–7). However, these medications require appropriate clinical assessment, monitoring, and consideration of eligibility, contraindications, and potential adverse effects. Gastrointestinal symptoms are among the most commonly reported adverse effects, and systematic reviews have also examined safety outcomes related to gallbladder, biliary, and pancreatitis-related events (8–10).

Beyond clinical effectiveness, the rapidly increasing public visibility of injectable weight-loss medications has raised important questions about how these treatments are understood outside formal healthcare settings. Online search trends indicate growing public interest in GLP-1 receptor agonists, particularly in high-income settings, raising broader public health concerns about access, equity, and public understanding of these treatments (11). Evidence from Great Britain has similarly highlighted growing public interest in the use of GLP-1 and GLP-1/GIP medications for weight loss, underscoring the need to examine how communities understand, consider, and use these treatments (12). In parallel, analyses of social media content suggest that semaglutide is often presented primarily as a weight-loss medication, with limited or inconsistent discussion of clinical indications, adverse effects, mechanisms of action, costs, and supply-related issues (13). Together, these findings highlight the need to examine how communities obtain information about weight-loss injections and how such information may shape their perceptions, interest, and potential use.

In Saudi Arabia, emerging evidence suggests growing public knowledge of GLP-1 receptor agonists and increasing reliance on social media as a source of information (14). However, previous Saudi studies have focused on specific populations or selected aspects of knowledge, attitudes, or medication use (14–16), leaving limited community-level evidence on how adults understand, perceive, use, or consider weight-loss injections outside formal clinical populations. This gap is important from a public health perspective because community exposure may shape perceptions of safety, effectiveness, appropriate use, and the need for medical supervision. The present study extends the available literature by providing a broader community-level assessment of how adults in Al-Ahsa understand, perceive, and engage with weight-loss injections.

Therefore, this study aimed to assess knowledge and attitudes regarding weight-loss injections among adults in Al-Ahsa, Saudi Arabia. It also aimed to identify the main sources of information, assess the perceived influence of social media, explore perceptions and common misconceptions regarding these medications, describe self-reported use or consideration of use, and examine reasons for use, discontinuation experiences, and willingness to recommend weight-loss injections. Furthermore, the study examined associations between weight-loss injection use or consideration and selected sociodemographic characteristics and BMI.

## Methodology

### Study design and setting

This descriptive, cross-sectional study assessed knowledge, attitudes, information sources, perceived social media influence, and self-reported use or consideration of weight-loss injections among adults in Al-Ahsa, Saudi Arabia.

### Study population and sampling

The target population comprised adult residents of Al-Ahsa, Saudi Arabia, aged 18 years and above. Participants were recruited using convenience sampling through WhatsApp, social media platforms, and personal networks. Eligibility criteria included being aged 18 years or older, residing in Al-Ahsa, Saudi Arabia, and providing voluntary informed consent.

Although convenience sampling was used, a minimum target sample size was estimated for descriptive analysis using the single-proportion formula. Based on an estimated population of approximately 1,300,000 residents in Al-Ahsa, a 95% confidence level, a 5% margin of error, and an expected prevalence of 50%, the minimum required sample size was 384 participants. The final sample included 718 eligible participants, exceeding the minimum estimated sample size.

Eligibility was assessed at the beginning of the questionnaire using screening questions concerning age and residence. To minimize duplicate submissions, the survey platform was configured to permit only one response per account. All 718 eligible responses were included in the final analysis. No responses were excluded because of ineligibility, questionnaire incompleteness, or potential duplication.

### Questionnaire and measures

Data were collected using a structured, self-administered questionnaire in Arabic. The questionnaire was developed by the authors based on the study objectives and informed by previous Saudi research examining public knowledge and attitudes toward weight-management medications (15), together with clinical evidence concerning the effectiveness and safety of GLP-1-based therapies (5, 6, 9, 10).

The final questionnaire comprised seven sections: (1) sociodemographic characteristics; (2) knowledge regarding weight-loss injections; (3) attitudes toward the safety and effectiveness of weight-loss injections; (4) perceived social media influence; (5) sources of information; (6) self-reported use or consideration of use; and (7) experiences among participants who reported using weight-loss injections.

In this study, knowledge refers to participants’ awareness of and information regarding weight-loss injections, including their indications, appropriate use, effectiveness, safety considerations, and potential adverse effects, as reflected in their responses to the questionnaire items. Attitudes refer to participants’ self-reported perceptions and evaluative views concerning the effectiveness, safety, medical supervision, and appropriate use of weight-loss injections. These measures reflect views regarding specific treatment-related issues rather than a comprehensive assessment of emotional disposition or actual medication-use behavior, which was assessed separately.

Knowledge and Awareness consisted of 11 items with response options of disagree, “do not know,” and agree. Perceptions and Attitudes consisted of five items rated on a five-point Likert scale ranging from strongly disagree to strongly agree. Because the items within these domains were phrased in different conceptual directions, they were analyzed descriptively at the item level rather than combined into an overall Knowledge or Attitude score.

The content validity of the questionnaire was assessed through expert review to evaluate the relevance, clarity, and appropriateness of the items in relation to the study objectives. The questionnaire was pilot-tested on a sample of 30 participants to assess clarity and comprehensibility. Internal consistency was also assessed using data from the pilot sample. Cronbach’s alpha coefficients were 0.769 for Knowledge and Awareness, 0.796 for Perceptions and Attitudes, and 0.823 for the Impact of Social Media, indicating acceptable to good internal consistency across the three domains.

Body mass index (BMI) was calculated using self-reported height and weight and categorized according to standard BMI classifications as underweight (<18.5 kg/m^2^), normal weight (18.5–24.9 kg/m^2^), overweight (25.0–29.9 kg/m^2^), and obesity (≥30.0 kg/m^2^). Analyses involving BMI were conducted using available cases; participants with missing BMI data were excluded only from BMI-related analyses.

### Statistical analysis

Data were analyzed using the Statistical Package for the Social Sciences (SPSS), version 29. Descriptive statistics were used to summarize participant characteristics, knowledge, attitudes, perceived social media influence, sources of information, and self-reported use patterns. Categorical variables and item-level responses for the Knowledge and Attitude domains are presented as frequencies and percentages.

Chi-square tests were used to examine associations between weight-loss injection use or consideration status and selected sociodemographic and BMI-related variables, including age group, sex, education level, and BMI category. Statistical significance was set at *p* < 0.05. Because the association analysis was exploratory and bivariate, the findings were interpreted as associations rather than independent predictors.

### Ethical considerations

Ethical approval was obtained from the Institutional Review Board at King Faisal University [KFU-REC-2024-MAY-ETHICS2406]. Participation was voluntary, and electronic informed consent was obtained before questionnaire completion. No personally identifiable information was collected, and all data were handled anonymously and confidentially.

## Results

A total of 718 participants were included in the analysis. Most participants were aged 18–25 years (60.3%), female (66.7%), and held a bachelor’s degree (67.5%). BMI data were available for 689 participants; among these, 41.9% were of normal weight, 26.3% were overweight, 23.1% were obese, and 8.7% were underweight (Table 1).

**Table 1:** Participant demographic characteristics.

Variables	Frequency	Percent (%)
Age		
18–25	433	60.3
26–35	88	12.3
36–45	73	10.2
46–55	50	7.0
56+	74	10.3
Sex		
Male	239	33.3
Female	479	66.7
Education level		
High school or below	126	17.5
Diploma	78	10.9
Bachelor’s degree	485	67.5
Master’s or higher	29	4.0
BMI categories (*n* = 689)^*^		
Underweight (<18.5)	60	8.7
Normal weight (18.5–24.9)	289	41.9
Overweight (25.0–29.9)	181	26.3
Obese (≥30.0)	159	23.1

^*^BMI data were unavailable for 29 participants; percentages are based on available data.

Most participants agreed that weight-loss injections require a medical prescription (71.9%) and work best when combined with diet and exercise (61.1%). More than half recognized nausea (59.2%), fatigue (62.1%), and digestive problems (50.3%) as common side effects (Table 2).

**Table 2:** Participants’ knowledge of weight-loss injections.

Items	Agree	Do not know	Disagree
	*n* (%)	*n* (%)	*n* (%)
Weight loss injections require a medical prescription.	516 (71.9)	133 (18.5)	69 (9.6)
Using weight loss injections is safe during pregnancy.	51 (7.1)	274 (38.2)	393 (54.7)
Weight loss injections work best when combined with diet and exercise.	439 (61.1)	204 (28.4)	75 (10.4)
Weight loss injections are a permanent solution for obesity.	91 (12.7)	188 (26.2)	439 (61.1)
Anyone can use weight loss injections regardless of their health condition.	66 (9.2)	133 (18.5)	519 (72.3)
Weight loss injections reduce appetite.	399 (55.6)	215 (29.9)	104 (14.5)
Weight loss injections increase metabolism (fat burning).	225 (31.3)	367 (51.1)	126 (17.5)
Weight loss injections increase appetite.	56 (7.8)	268 (37.3)	394 (54.9)
Common side effect: fatigue.	446 (62.1)	209 (29.1)	63 (8.8)
Common side effect: digestive problems	361 (50.3)	285 (39.7)	72 (10.0)
Common side effect: nausea.	425 (59.2)	234 (32.6)	59 (8.2)

Knowledge gaps were also observed. More than half of the participants were unsure whether weight-loss injections increase metabolism or fat burning (51.1%). In addition, 29.9% were unsure whether these injections reduce appetite, and 39.7% were unsure whether digestive problems are common side effects.

Most participants agreed or strongly agreed that using weight-loss injections without medical consultation can be dangerous (77.3%). In contrast, 56.3% disagreed or strongly disagreed that weight-loss injections are safe for long-term use, while 57.4% disagreed or strongly disagreed that these injections eliminate the need for diet and exercise (Table 3).

**Table 3:** Participants’ attitudes toward weight loss injections.

Items	Strongly agree	Agree	Neutral	Disagree	Strongly disagree
	*n* (%)	*n* (%)	*n* (%)	*n* (%)	*n* (%)
I believe weight loss injections are an effective method for weight control.	93 (13.0)	148 (20.6)	261 (36.4)	164 (22.8)	52 (7.2)
I believe weight loss injections are safe for long-term use.	63 (8.8)	62 (8.6)	189 (26.3)	283 (39.4)	121 (16.9)
Using weight loss injections without medical consultation can be dangerous.	406 (56.5)	149 (20.8)	87 (12.1)	50 (7.0)	26 (3.6)
I believe using weight loss injections eliminates the need for diet and exercise.	54 (7.5)	91 (12.7)	161 (22.4)	283 (39.4)	129 (18.0)
I believe weight loss injections provide a quick and effective solution for obesity.	97 (13.5)	169 (23.5)	252 (35.1)	136 (18.9)	64 (8.9)

Responses were more mixed regarding the perceived effectiveness of weight-loss injections. Overall, 36.4% of participants expressed a neutral view regarding whether weight-loss injections are effective for weight control, while 35.1% were neutral regarding whether these injections provide a quick and effective solution for obesity. These findings indicate that participants generally expressed caution regarding unsupervised use and long-term safety, while perceptions of effectiveness were less clearly defined.

Participants reported mixed perceptions of social media content related to weight-loss injections. Overall, 42.3% reported that social media made them more skeptical about weight-loss injections, while 35.7% believed that it spreads misleading information. In contrast, 40.3% believed that social media provides accurate and useful information, whereas 25.6% reported that exposure to social media increased their confidence in the effectiveness and safety of weight-loss injections (Table 4).

**Table 4:** Participants’ perceptions of social media content related to weight-loss injections.

Items	Yes	Neutral	No
	*n* (%)	*n* (%)	*n* (%)
Social media increased my confidence in the effectiveness and safety of weight loss injections.	184 (25.6)	245 (34.1)	289 (40.3)
Social media made me more skeptical about weight loss injections.	304 (42.3)	217 (30.2)	197 (27.4)
Social media provided me with accurate and useful information.	289 (40.3)	226 (31.5)	203 (28.3)
Social media spread misleading information.	256 (35.7)	307 (42.8)	155 (21.6)
Social media had no impact on my opinion.	262 (36.5)	235 (32.7)	221 (30.8)

Social media was the most commonly reported source of information about weight-loss injections (62.1%), followed by friends or family (41.5%) and doctors or other healthcare professionals (39.1%). Advertisements were reported as a source of information by 16.4% of participants, while 4.6% reported obtaining information from other sources.

Overall, 61 participants (8.5%) reported current or previous use of weight-loss injections, while another 61 participants (8.5%) reported considering future use. Among participants who reported using weight-loss injections, tirzepatide was the most commonly reported injection (42.6%), followed by semaglutide (39.3%) and liraglutide (14.8%). Most users reported using weight-loss injections for 3–6 months (41.0%), followed by less than 3 months (31.1%) (Table 5).

**Table 5:** Self-reported use, consideration, and characteristics of weight-loss injection use.

Variables	Frequency	Percent (%)
Use of weight loss injections		
Yes	61	8.5
No	596	83.0
Considering	61	8.5
Participants who reported using weight-loss injections (*n* = 61)		
Type of injection you used		
Liraglutide (Saxenda)	9	14.8
Tirzepatide (Mounjaro)	26	42.6
Semaglutide (Ozempic)	24	39.3
Other	2	3.3
Duration of use		
Less than 3 months	19	31.1
3 to 6 months	25	41.0
6 months to 1 year	8	13.1
More than 1 year	9	14.8
Medical reasons for use		
Obesity (Body Mass Index > 30)	23	37.7
Overweight with comorbidities (e.g., high blood pressure or cholesterol)	8	13.1
Type 2 diabetes	13	21.3
Metabolic disorder (e.g., PCOS or insulin resistance)	5	8.2
No medical condition—used injections for weight loss only	12	19.7
Reasons for initiating weight-loss injections		
Direct medical advice from a doctor due to a health condition.	14	23.0
Personal decision to lose weight faster than with diet and exercise alone.	23	37.7
Influenced by social opinions or pressure (from friends, family, or social media).	6	9.8
Previous unsuccessful weight loss attempts.	16	26.2
To maintain weight after losing it.	2	3.3
Pattern of injection use		
Used injections as the main method for weight loss.	28	45.9
Used injections with diet only.	13	21.3
Used injections with exercise only.	6	9.8
Used injections with both diet and exercise.	12	19.7
Other	2	3.3
Total weight lost during injection use		
Did not lose weight	6	9.8
Less than 5 kg	14	23.0
5–10 kg	17	27.9
10–15 kg	16	26.2
15 kg or more	8	13.1

Regarding reported medical reasons for use, 37.7% of users reported obesity, 21.3% reported type 2 diabetes, 13.1% reported overweight with comorbidities, and 8.2% reported a metabolic disorder. In addition, 19.7% reported using weight-loss injections for weight loss without a reported medical condition.

The most frequently reported reason for using weight-loss injections was the desire to lose weight faster than with diet and exercise alone (37.7%), followed by previous unsuccessful weight-loss attempts (26.2%) and direct medical advice from a doctor because of a health condition (23.0%). A smaller proportion reported being influenced by social opinions or pressure from friends, family, or social media (9.8%).

Regarding patterns of use, 45.9% reported using weight-loss injections as their main method of weight loss, while 21.3% used them with diet only, 9.8% with exercise only, and 19.7% with both diet and exercise. Reported weight loss varied among users: 27.9% reported losing 5–10 kg, 26.2% reported losing 10–15 kg, 13.1% reported losing 15 kg or more, and 9.8% reported no weight loss.

Among participants who had used weight-loss injections, 55.7% were still using them at the time of the survey, while 44.3% had discontinued use. Among those who had discontinued treatment, the most commonly reported reason was inability to tolerate side effects (37.0%), followed by reaching the desired weight (25.9%) and lack of perceived benefit (11.1%). When asked whether they would recommend weight-loss injections to others, the most frequent response was that the recommendation would depend on the individual’s medical condition (26.2%) (Table 6).

**Table 6:** Ongoing use and discontinuation of weight-loss injections (*n* = 61).

Variables	Frequency	Percent (%)
Are you still using weight loss injections? (*n* = 61)		
Yes	34	55.7
No	27	44.3
Reasons for stopping the use of weight loss injections (*n* = 27)		
Reached desired weight	7	25.9
Could not tolerate side effects	10	37.0
Did not benefit from the injections	3	11.1
Underwent bariatric surgery (e.g., sleeve, balloon)	1	3.7
Other (Unavailable previously; boredom; family concern; used initially as motivation, stopped after reaching goal weight; continuing exercise and diet.)	6	22.2
Would you recommend weight loss injections to others? (*n* = 61)		
Strongly yes	14	23.0
Yes	11	18.0
Neutral	12	19.7
No	6	9.8
Strongly no	2	3.3
Depends on the medical condition	16	26.2

Exploratory bivariate analysis showed that weight-loss injection use or consideration status was significantly associated with age group (*χ*^2^ = 48.291, *p* < 0.001), education level (*χ*^2^ = 13.770, *p* = 0.032), and BMI category (*χ*^2^ = 67.048, *p* < 0.001). No significant association was observed between sex and weight-loss injection use or consideration status (*χ*^2^ = 1.815, *p* = 0.403).

## Discussion

This study assessed knowledge, attitudes, information sources, perceived social media influence, and self-reported use or consideration of weight-loss injections among adults in Al-Ahsa, Saudi Arabia. Overall, the findings indicate considerable uncertainty across several knowledge items, mixed perceptions regarding the effectiveness and safety of weight-loss injections, relatively low current or previous use, emerging interest in future use, and substantial reliance on social media as a source of information. These findings provide insight into how weight-loss injections are understood and perceived at the community level and highlight areas where improved medication literacy and appropriate clinical communication may be needed.

Knowledge findings showed a mixed pattern, with good recognition of some safety-related aspects but considerable uncertainty regarding several other aspects of weight-loss injections. Participants showed relatively good recognition that these medications require medical supervision and that their use is most appropriately integrated with dietary and physical activity interventions. However, important gaps remained regarding their mechanisms of action and potential adverse effects. This pattern suggests that general familiarity with weight-loss injections may be developing faster than detailed medication literacy. This distinction is important because GLP-1 receptor agonists and related agents are medically indicated therapies that require appropriate patient selection, assessment of eligibility, monitoring, and ongoing lifestyle support (17). Similar gaps in public knowledge have been reported in previous Saudi studies examining GLP-1 receptor agonists and weight-management medications (14, 15). Given the increasing clinical use of semaglutide and tirzepatide and evidence of clinically meaningful weight reduction among eligible adults with overweight or obesity (5, 6), public health education should focus not only on increasing awareness of these medications but also on improving understanding of their indications, expected benefits, limitations, potential adverse effects, and the importance of professional supervision.

Participants’ responses reflected a cautious but mixed view of weight-loss injections. There was substantial recognition of the risks of using these medications without medical consultation and concern regarding long-term safety, while perceptions of their effectiveness were less consistent. This pattern may reflect uncertainty about the appropriate role of these medications in weight management rather than uniform acceptance or rejection. Similar ambivalence has been reported in community-based research on GLP-1 medications for weight loss, where perceived benefits coexist with concerns regarding safety, accessibility, and broader implications of pharmacological weight management (18). Patient-experience research has likewise shown that users may weigh perceived weight-loss benefits against adverse effects, treatment burden, and concerns regarding treatment continuation or discontinuation (19). These findings suggest that public health communication should focus on informed and appropriate use while providing realistic information regarding expected benefits, potential risks, and treatment duration.

The prevalence of self-reported current or previous use of weight-loss injections in this study was relatively low, while a similar proportion of participants reported considering future use. This pattern suggests that, within this community sample, weight-loss injections were not yet widely used but had become an emerging area of public interest. The distinction between current use and future consideration is relevant for public health planning because interest may increase as these medications become more visible and accessible. A population-based study in Great Britain similarly found that although use of GLP-1 and GLP-1/GIP medications for weight loss remained limited at the population level, interest in future use was evident among non-users (12).

The findings should also be considered within the broader Gulf regional context. Recent real-world evidence from the United Arab Emirates demonstrated a substantial increase in the use of GLP-1-based therapies between 2022 and 2024, together with a marked shift toward tirzepatide and heterogeneous weight responses (20). Although this clinical population differs from the community-based sample in the present study, the UAE findings demonstrate that the increasing visibility of GLP-1-based therapies is accompanied by growing integration into routine clinical practice within the Gulf region. Together, these findings support the importance of individualized treatment, appropriate monitoring, patient counseling, and further regional research examining real-world use and outcomes.

Social media emerged as the most frequently reported source of information about weight-loss injections, exceeding healthcare professionals. This finding highlights the important role of digital platforms in shaping public exposure to GLP-1-based therapies and may partly explain the increasing visibility of these medications outside clinical settings. At the same time, social media may create an information environment in which benefits, risks, eligibility, and appropriate monitoring are presented inconsistently. Previous Saudi evidence has similarly suggested that public knowledge of weight-management medications remains incomplete despite recognition of commonly discussed agents such as semaglutide, with increasing exposure to information through non-clinical channels (14, 15). International analyses of semaglutide and GLP-1 receptor agonist content have also shown that social media posts and advertisements may emphasize weight loss, convenience, and emotional appeal while providing limited or inconsistent information regarding clinical indications, adverse effects, mechanisms of action, costs, and supply-related concerns (13, 21). In the present study, participants’ perceptions reflected this complexity: social media was not uniformly perceived as either beneficial or harmful, with some participants reporting increased confidence while others reported skepticism or concern regarding misleading information. Thus, social media should be considered an important but potentially inconsistent source of health information, capable of increasing awareness and interest while also influencing expectations and perceptions regarding the safety and appropriateness of weight-loss injections.

These findings also have implications for appropriate access to and safe use of weight-loss injections. Although most participants recognized that these medications require a medical prescription, a notable minority were uncertain or disagreed, indicating incomplete awareness of appropriate access requirements. Because the present study did not assess medication-access pathways, dispensing practices, or regulatory compliance, it cannot determine whether additional regulatory restrictions are required. Nevertheless, the findings support reinforcing prescription-based dispensing, appropriate assessment of treatment eligibility, and medical supervision before and during treatment (17, 22). Pharmaceutical and promotional materials should likewise provide balanced and sufficiently comprehensive information to support informed decision-making, particularly given evidence that social media advertisements may emphasize benefits while providing inconsistent information about risks and appropriate use (13, 21).

Beyond appropriate access and information, the reported pattern of use also raises an important issue regarding the role of lifestyle modification. Some participants reported using weight-loss injections as their main method of weight loss, whereas fewer reported combining them with both diet and exercise. This finding is important because pharmacological treatment is intended to form part of comprehensive obesity management rather than replace health behavior interventions. Current obesity pharmacotherapy guidance emphasizes individualized treatment combined with appropriate nutrition and physical activity (22). Recent evidence also suggests that combining GLP-1 receptor agonists with lifestyle modification may improve weight-related and cardiometabolic outcomes among adults with overweight or obesity, although the certainty of evidence varies across outcomes and intervention characteristics (23). Evidence on weight maintenance further supports the role of exercise, with improvements in body weight and composition following GLP-1 receptor agonist treatment being better sustained when exercise was incorporated (24). Therefore, the reported reliance on injections as a primary weight-loss method among some participants may indicate a gap between recommended comprehensive obesity care and community practices. Public health and clinical counseling should consequently emphasize that pharmacological treatment is one component of weight management and should be accompanied by appropriate dietary counseling, physical activity, monitoring, and follow-up.

Among participants who had used weight-loss injections, a substantial proportion had discontinued treatment, with inability to tolerate side effects being the most commonly reported reason for discontinuation. This finding highlights the importance of tolerability in sustained pharmacological weight management. Although GLP-1 receptor agonists and related therapies have demonstrated clinically meaningful weight-loss effects, gastrointestinal and other adverse effects may affect treatment continuation, adherence, and patient satisfaction (8, 25). Real-world evidence also indicates that discontinuation of GLP-1 receptor agonists is common among adults with overweight or obesity, with patterns influenced by clinical status and treatment context (26). Available Saudi evidence remains limited to selected clinical and patient samples. A Saudi study reported gastrointestinal adverse events among patients receiving GLP-1 receptor agonists at three tertiary centers in Riyadh (27). Another Saudi study documented adverse-effect reporting and safety concerns among previous anti-obesity medication users attending hospitals in Jeddah (28). Although these studies provide relevant national evidence, their findings cannot establish population-level complication rates or estimates specific to Al-Ahsa.

In the present study, the most frequent recommendation response was that use ‘depends on the medical condition,’ which may reflect recognition that these medications are not universally suitable for all individuals. This finding reinforces the importance of individualized assessment, pre-treatment counseling, adverse-effect management, follow-up, and realistic expectations regarding treatment duration and continuation. Evidence regarding weight regain after treatment cessation further supports the importance of longer-term weight-management planning rather than viewing pharmacological treatment as a short-term intervention (29).

The exploratory bivariate analysis showed that use or consideration of weight-loss injections differed by age group, education level, and BMI category, but not by sex. These findings should be interpreted cautiously because the analysis was cross-sectional and unadjusted, and the number of participants reporting current or previous use was relatively small. Therefore, these associations should not be interpreted as independent predictors of use or consideration. The observed association with BMI is clinically plausible, as individuals with overweight or obesity may be more likely to seek or consider pharmacological weight-management options. Similar population-based evidence has demonstrated variation in the use of and interest in GLP-1 receptor agonists according to demographic and weight-related characteristics (12). Future studies using representative samples and multivariable analyses are needed to determine whether these characteristics independently influence the use, consideration, or accessibility of weight-loss injections.

Taken together, these findings underscore the need to strengthen medication literacy surrounding weight-loss injections. Public education should move beyond simple awareness of the availability of these treatments and provide clear information regarding appropriate eligibility, realistic treatment benefits, potential adverse effects, and the importance of clinical monitoring. The prominent role of social media in the present study further emphasizes the need for healthcare professionals and public health organizations to provide accessible, evidence-based information through channels commonly used by the public. Such communication should support informed decision-making and reinforce that pharmacological treatment should be integrated with appropriate nutrition, physical activity, and follow-up care.

## Strengths and limitations

This study addresses a timely public health issue and provides community-level evidence on knowledge, attitudes, information sources, perceived social media influence, and self-reported use or consideration of weight-loss injections among adults in Al-Ahsa, Saudi Arabia. A further strength is that the study examined not only current or previous use but also future consideration, patterns of use, discontinuation, and willingness to recommend these medications, providing a broader view of how weight-loss injections are perceived and used outside formal clinical settings.

This study also has several limitations. The use of convenience sampling and online recruitment may limit the generalizability of the findings and may have resulted in the overrepresentation of younger, more educated, or more digitally active participants. The data were self-reported, which may have introduced recall, reporting, or misclassification biases, particularly regarding medication type, duration of use, weight loss, reasons for discontinuation, and BMI. The relatively small number of participants who reported current or previous use of weight-loss injections limited the precision of findings related to users’ experiences and discontinuation. In addition, the cross-sectional design does not allow causal inferences to be made. Although the questionnaire was developed based on relevant literature, reviewed by experts, and pilot-tested for clarity and comprehensibility, its psychometric properties require further evaluation. In the pilot sample, Cronbach’s alpha coefficients ranged from 0.769 to 0.823 across the three domains; however, formal psychometric validation beyond expert review and internal consistency assessment was not performed.

A further limitation relates to the assessment of treatment-related complications. Although participants were asked about reasons for discontinuing weight-loss injections, including an inability to tolerate side effects, the questionnaire was not designed to systematically ascertain or clinically verify treatment-related complications. Information regarding the nature, severity, timing, and clinical attribution of specific adverse events was therefore unavailable, precluding estimation of their incidence or prevalence in Al-Ahsa. Furthermore, the available Saudi evidence is derived primarily from selected clinical or patient samples rather than population-based surveillance. Further epidemiological and pharmacovigilance studies are needed to characterize the frequency, severity, and clinical implications of adverse outcomes associated with weight-loss injections in Saudi Arabia.

## Conclusion

This study found that adults in Al-Ahsa showed considerable uncertainty across several knowledge items and mixed perceptions regarding the effectiveness and safety of weight-loss injections. Although current or previous use was relatively low, the proportion considering future use suggests emerging public interest. Social media was the most frequently reported source of information, exceeding healthcare professionals, while participants’ perceptions of social media content were mixed. These findings highlight the need for evidence-based public health communication, stronger involvement of healthcare professionals in medication education, and medication safety messages that promote appropriate medical consultation, realistic expectations, and integration of pharmacological treatment with dietary practices and physical activity. Future research using representative sampling and longitudinal or mixed-methods designs is needed to better understand changing public knowledge, attitudes, access, and use patterns, as well as the real-world safety and complications associated with weight-loss injections.

## Data Availability

The raw data supporting the conclusions of this article will be made available by the authors, without undue reservation.
